# Loop-mediated isothermal amplification (LAMP) assay for identification of Australian Plague Locust (APL), *Chortoicetes terminifera* (Walker, 1870)

**DOI:** 10.1038/s41598-026-50241-7

**Published:** 2026-05-21

**Authors:** Lea Rako, Francesco Martoni, Cait Selleck, Michael R. Kearney, Brendan C. Rodoni, Mark J. Blacket

**Affiliations:** 1 Agriculture Science and Technology, AgriBio-Centre for AgriBioscience, 5 Ring Road, Bundoora, VIC 3083 Australia; 2 School of Applied Systems Biology, La Trobe University, Bundoora, VIC 3083 Australia; 3 Climatic and Metabolic Ecology Lab (CAMEL), University of Melbourne, Parkville, VIC 3010 Australia

**Keywords:** Molecular APL identification, Cytochrome b, Genie III, Field diagnostics, Pest management, Biosecurity, Biological techniques, Biotechnology, Ecology, Ecology, Plant sciences, Zoology

## Abstract

The Australian Plague Locust (APL), *Chortoicetes terminifera*, the most important pest locust in Australia, can cause significant damage to crops and grasslands when present in high numbers. We have developed a new LAMP (loop-mediated isothermal amplification) assay specifically targeting APL for rapid diagnostics to support locust management. We tested five DNA extraction methods for potential near-field use and designed an APL gBlock as a synthetic positive control. The performance of new LAMP assay was assessed against a panel of eleven closely related Australian grasshopper species (family Acrididae). The optimised LAMP assay produced amplification only from APL DNA, on average in 18.0 ± 2.9 min, with an anneal derivative of 79.9 ± 0.5 °C, and gBlock produced a different anneal derivative of 83.5 ± 0.5 °C. The LAMP assay was sensitive to very low levels of DNA, with detection down to 0.2 picogram of APL DNA. The speed of application and accurate identification results generated, enables this scalable assay to be easily used in the field as a new tool for APL control, providing positive/negative results within an hour. This capability will prove an essential aid during sporadic APL population growth events, when accurate species-level identification of substantial numbers of nymphs, prior to adult locusts swarming, is a critical aspect of pest management.

**Supplementary Information:**

The online version contains supplementary material available at 10.1038/s41598-026-50241-7.

## Introduction

Locusts are grasshopper insects in the order Orthoptera, which differ by their behavioural ability to swarm under favourable conditions^1^. The Australian Plague Locust (APL), *Chortoicetes terminifera* (Walker, 1870), the most important pest species of locust in Australia, is capable of sporadic swarming and infesting large areas of open grassland and crops under favourable environmental conditions^1–3^. In the event of a regional APL outbreak it is of utmost importance to be able to identify early instar grasshoppers (flightless nymphs) before adult locusts start to swarm, to enable pest control prior to swarms causing extensive economic crop damage, and rapid migration of adult APL to new areas^1,4–6^. When APL outbreaks occur, vast numbers of grasshopper samples, at all development stages including nymphs, are commonly submitted to specialised diagnostic laboratories for morphological identification. These identifications require high-level entomological expertise, with identification of specimens to species-level by highly trained entomologists being extremely time consuming^1,5^. This time delay can lead to ineffective swarm management responses, which rely on control measures being applied prior to adult locusts dispersing^1,3,5^.

**Fig. 1:**
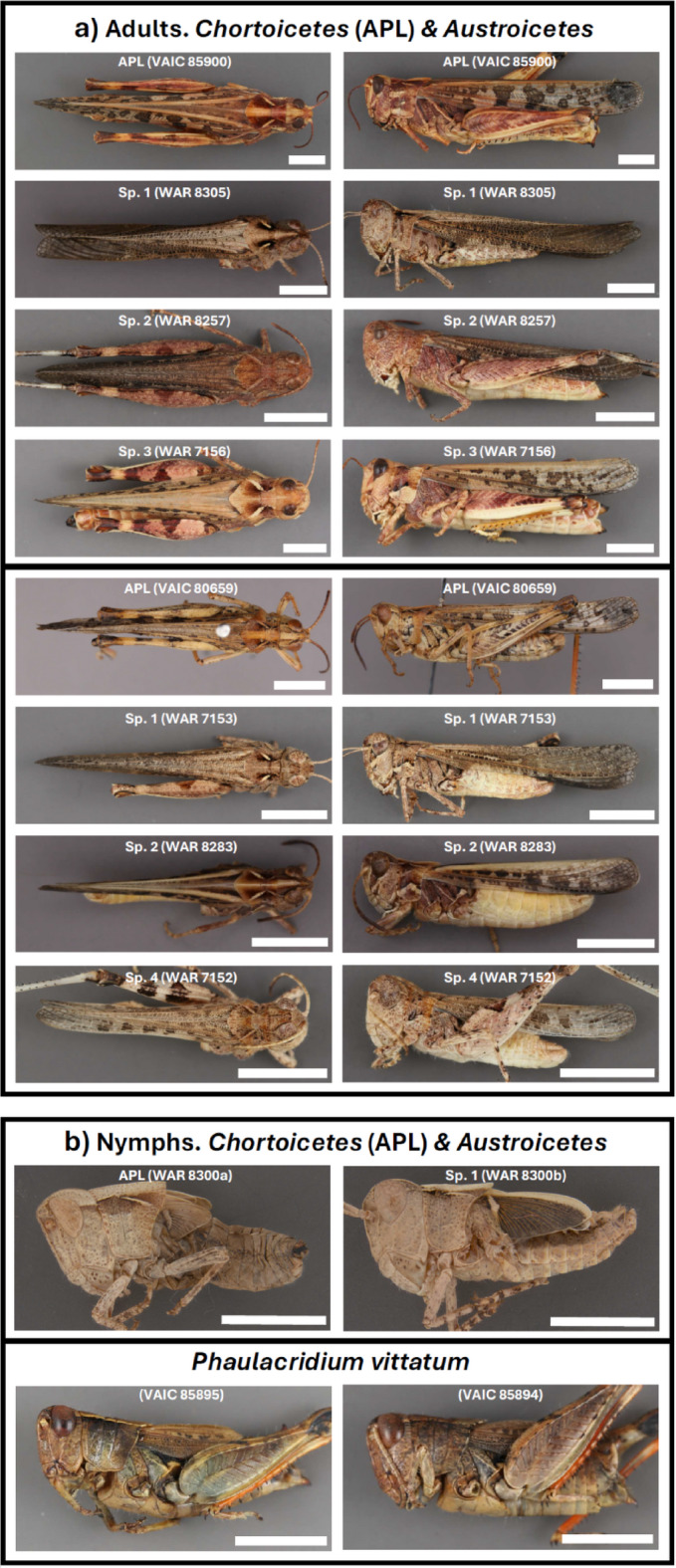
*Chortoicetes terminifera* (APL) and *Austroicetes* (Sp. 1 to Sp. 4) used to develop the LAMP assay. (**a**) Adults. Females upper, males lower. (**b**) Nymphs. Morphological comparison of similar sized juvenile *Chortoicetes*, *Austroicetes*, and *Phaulacridium* specimens. White scale-bars indicate 5 mm. All specimens identified through DNA barcoding (see below). VAIC, Victorian Agricultural Invertebrate Collection; WAR, Specimens collected by M. Kearney. Specimens were photographed by Cait Selleck.

Many closely related Australian grasshoppers (Orthoptera, Acrididae) appear morphologically similar to APL, especially the closest relatives in the genus *Austroicetes* (Fig. 1), which commonly co-occur with APL^1,5^. Identification of immature APL is highly problematic as many Australian grasshopper species appear morphologically similar and are especially difficult to differentiate as immature nymphs^5^(Fig. 1). Current molecular diagnostic identification of grasshopper species relies on DNA barcoding^7–9^ to provide species level identifications. However, this process is slow in the context of locust swarm development, i.e., requiring days, as it involves the generation of DNA sequences to provide identification, making it relatively expensive and requiring specialized laboratory facilities and trained staff. An alternative much more rapid diagnostic approach is loop-mediated isothermal amplification (LAMP)^10^, a technique which utilises constant-temperature amplification to provide results within one hour and has in recent years seen technological advances and more widespread application. LAMP assays have previously been developed for diagnosing many plant pests in the laboratory and field including, Queensland fruit fly *Bactrocera tryoni*^11^, grape phylloxera *Daktulosphaira vitifoliae*^12^, Khapra beetle *Trogoderma granarium*^13^, Fall armyworm *Spodoptera frugiperda*^14^, and *Varroa destructor* mite^15^.

In the current study we develop and test a new LAMP assay to enable quick molecular diagnosis of APL, *Chortoicetes terminifera*. The primary aims of the current study were to: develop and optimise a new, rapid and reliable molecular diagnostic LAMP assay for identification of APL, test DNA extraction methods suitable for near-field use, design a gBlock gene fragment to be used as synthetic positive control, and test the specificity of new LAMP assay for detection of APL against a panel of closely related (Acrididae) Australian grasshopper species.

## Results

### APL LAMP assay design and optimisation

**Fig. 2:**
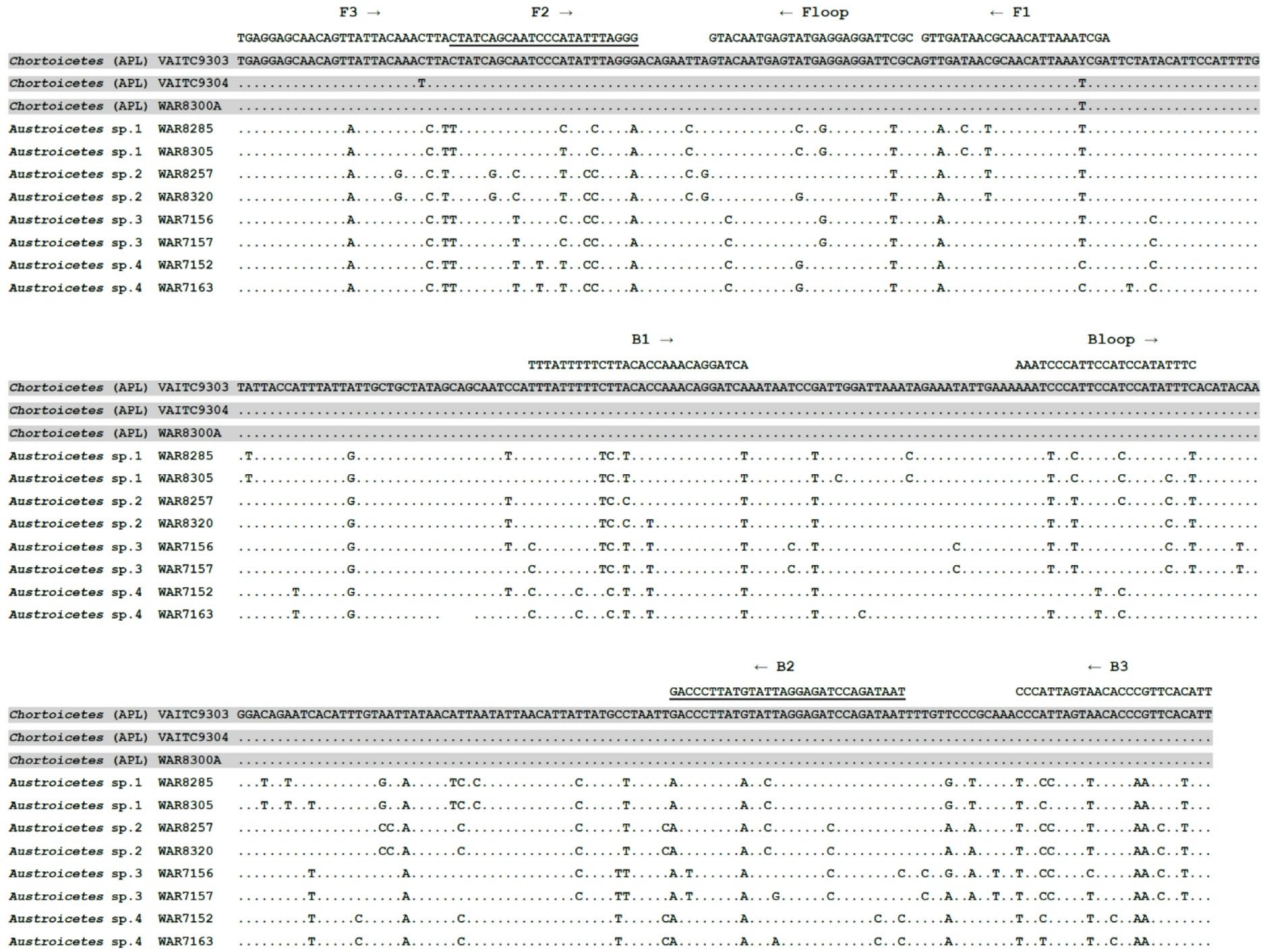
DNA sequence alignment of the mitochondrial Cytb locus from *Chortoicetes terminifera* (APL), and four *Austroicetes* species (Sp. 1 to Sp. 4), used for primer design of the LAMP assay. Grey shading highlights APL. LAMP primers are indicated above the alignment, with F2 and B2 underlined.

**Fig. 3:**
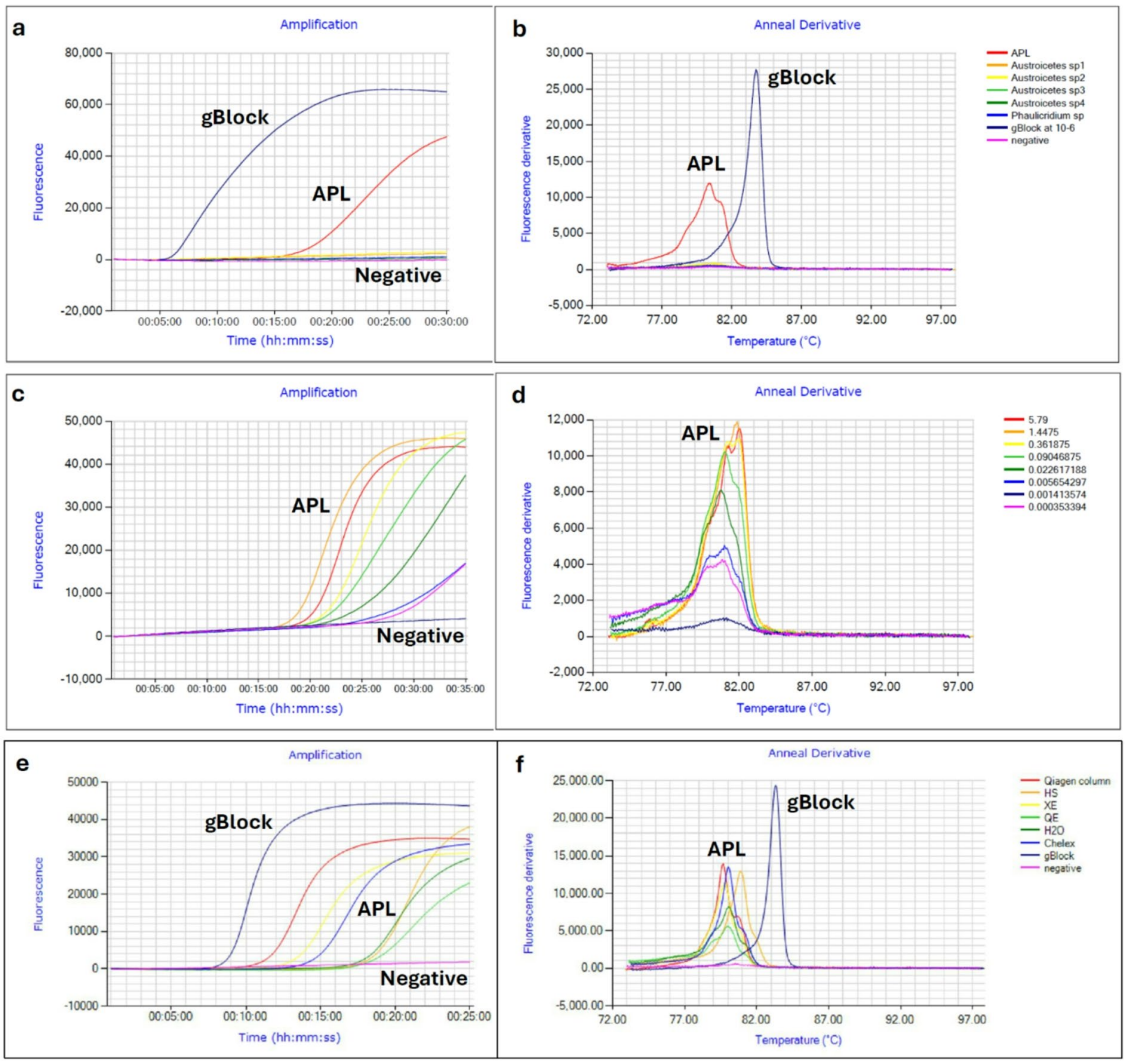
Optimised APL LAMP assay results. (**a**) LAMP amplification of *Chortoicetes terminifera* (APL) DNA, synthetic positive gBlock and non-target “negative” DNA samples. (**b**) Anneal derivative of (**a**), showing difference between APL DNA and gBlock. (**c**) Dilution series of APL DNA. (**d**) Anneal derivative of APL DNA. (**e**) LAMP amplification of alternative DNA extractions of VAITC 9713, (**f**) Anneal derivate of (**e**).

We developed six LAMP primers for a new APL LAMP assay (Fig. 2; Table 1). The optimised assay produced amplification of the APL sample, on average (*n* = 37) in 18.0 ± 2.9 min, with an anneal derivative of 79.9 ± 0.5 °C (Fig. 3a, b). Some of the archived APL DNA samples (*n* = 7) produced anneal derivate temperatures, but failed to record amplification times, as the DNA extractions appeared to be degraded through storage, even though they had been previously sequenced for DNA barcoding identification approximately five years prior.

**Table 1:** Australian Plague Locust (APL), *Chortoicetes terminifera*, Cytb LAMP primer and amplicon (gBlock) sequences and parameters. The F2 and B2 primer regions of the FIP and BIP primers are in bold. Lowercase letters in the gBlock indicate extra “cctcc”s added between LAMP primer sites to increase the overall Tm of the amplicon.

Primer	Sequence 5–3′	Primer Length, bp	Predicted Tm, °C	GC %
APL_F3	TGAGGAGCAACAGTTATTACAAACTTA	27	63.1	33.3
APL_B3	AATGTGAACGGGTGTTACTAATGGG	25	67.5	44.0
APL_Floop	GCGAATCCTCCTCATACTCATTGTAC	26	66.8	46.2
APL_Bloop	AAATCCCATTCCATCCATATTTC	23	63.8	34.8
APL_FIP	CGATTTAATGTTGCGTTATCAAC*CTATCAGCAATCCCATATTTAGGG*	47	79.3	38.3
APL_BIP	TTTATTTTTCTTACACCAAACAGGATCAA*TTATCTGGATCTCCTAATACATAAGGGTC*	58	77.9	32.8
APL gBlock sequence	cctccTGAGGAGCAACAGTTATTACAAACTTAcctccCTATCAGCAATCCCATATTTAGGGcctccGTACAATGAGTATGAGGAGGATTCGCcctccGTTGATAACGCAACATTAAATCGAcctccTTTATTTTTCTTACACCAAACAGGATCAcctccAAATCCCATTCCATCCATATTTCcctccGACCCTTATGTATTAGGAGATCCAGATAATcctccCCCATTAGTAACACCCGTTCACATTcctcc	252	N/A	N/A

### APL LAMP assay specificity results

**Fig. 4:**
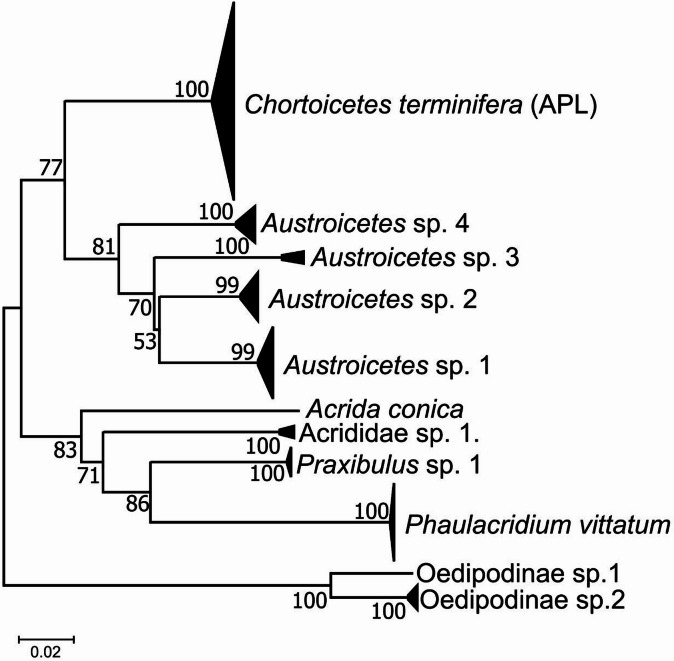
Neighbour Joining tree showing the relationships between *Chortoicetes terminifera* (APL) and closely related genera within the family Acrididae, from 87 *Cytochrome b* DNA sequences generated in the current study.

We assessed the performance of new LAMP assay against a panel of closely related Australian grasshopper species (family Acrididae) (Table 2). DNA barcoding of cytochrome b (Cytb)^7^ was used to delimit and identify taxa confirming the species identification of grasshopper samples (Fig. 4, Sup. Table). All 37 specimens of APL tested produced positive amplification for the APL LAMP assay, while the 56 non-target grasshoppers (10 x species) did not amplify (Table 2, Sup. Table).

**Table 2:** List of the 11 taxa tested for this work. Each taxon is reported with the number of individuals tested, taxonomic identification, and GenBank accession numbers. Target species has been highlighted.

Family	Genus	Species	Individuals	GenBank accessions
Acrididae	*Acrida*	*Acrida conica*	1	PX315616
Acrididae	*Austroicetes*	*Austroicetes* sp. 1	12	PX315617 - PX315628
Acrididae	*Austroicetes*	*Austroicetes* sp. 2	8	PX315629 - PX315636
Acrididae	*Austroicetes*	*Austroicetes* sp. 3	2	PX315637 - PX315638
Acrididae	*Austroicetes*	*Austroicetes* sp. 4	6	PX315639 - PX315644
Acrididae	*Chortoicetes*	*Chortoicetes terminifera*	34	PX315645 - PX315678
Acrididae	Undetermined	Acrididae sp. 1	2	PX315679 - PX315680
Acrididae	Undetermined	Oedipodinae sp. 2	3	PX315681 - PX315683
Acrididae	Undetermined	Oedipodinae sp. 1	1	PX315684
Acrididae	*Phaulacridium*	*Phaulacridium vittatum*	13	PX315685 - PX315697
Acrididae	*Praxibulus*	*Praxibulus* sp. 1	5	PX315698 - PX315702

### APL LAMP assay sensitivity test

**Fig. 5:**
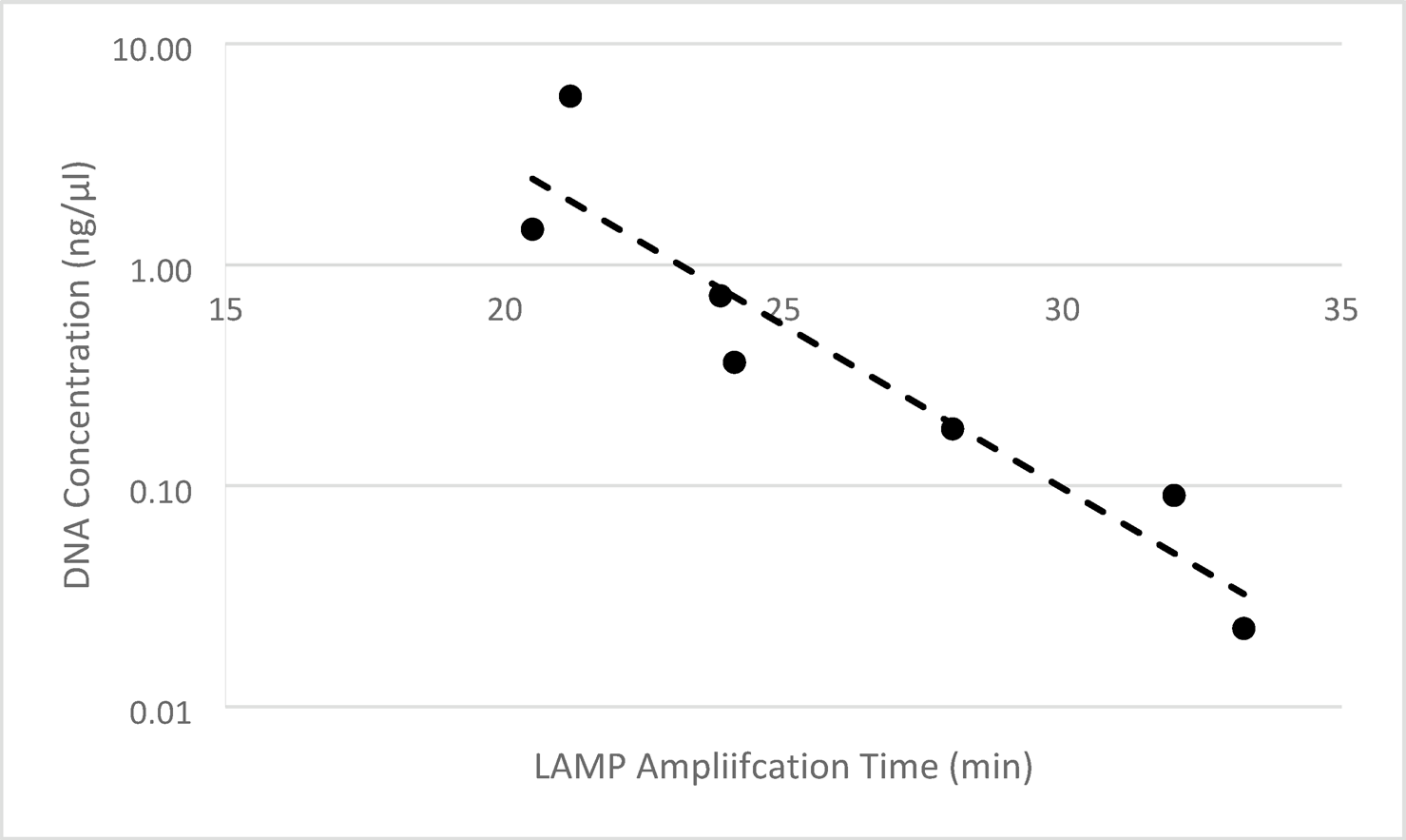
APL LAMP sensitivity test for a four-fold serial dilution of DNA extracts from *Chortoicetes terminifera* (APL), sample VAITC 9303. Amplification times are shown for DNA sample with DNA concentration ranging from highest (5.8 ng/µl,) to lowest (0.02 ng/µl) amount, amplifying in 21.2 to 33.3 min respectively. Exponential regression line, R^2^ = 0.46.

Sensitivity of the LAMP assay was assessed through a four-fold serial dilution of APL DNA which produced positive LAMP amplifications over eight DNA dilutions (Fig. 5). The LAMP assay was found to be very sensitive and amplified low levels of APL DNA, with decreasing amounts of DNA producing longer amplification times (Fig. 5). The highest DNA concentration 5.8 ng/µL produced amplification in 21.2 min and the lower DNA concentration 0.02 ng/µl amplified in 33.3 min.

### Evaluation of APL gBlock gene fragment

Amplification of the 252 bp APL gBlock was very sensitive, being detected as low as ~ 1 × 10^1^ copies/µL of APL gBlock within 10.1 min with an anneal derivative of 83.5 ± 0.5 °C. All dilutions amplified within 5.2 to 10.1 min, much quicker than the clean laboratory prepared DNA extraction of APL specimen VAITC 9303 which we used as a standard sample for developing this test. One million copies (1 × 10^6^) of gBlock amplified within an average of 8.8 min (*n* = 15 runs). Based on this amplification time, 1 × 10^6^ copies/µL of APL gBlock was found to be suitable for use as synthetic positive in APL LAMP assay. The anneal derivative of LAMP amplicons produced two distinct peaks, 79.5 ± 0.5 °C for APL genomic DNA and 83.3 ± 0.5 °C for the APL gBlock which, with 3 °C difference were easily distinguishable from each other (Fig. 3c, d).

### Evaluation of field-appropriate DNA extraction methods

We tested six different DNA extraction methods to assess which would be appropriate for near-field use. All tests were performed on a single specimen VAITC 9713 (APL nymph), (Table 3; Fig. 3e, f). The standard Qiagen (column) extraction method produced clean and pure laboratory grade DNA, which is ideal for long-term storage, and produced on average the quickest LAMP amplification times (13.7 ± 0.8 min). As expected, the ddH_2_O (water) gave the slowest amplification times (17.2 ± 2.1 min).

**Table 3:** Comparison of different laboratory-based and near-field compatible DNA extraction methods used in APL LAMP assay. APL sample = VAITC 9713, *n* = 7 replicates tested for each method, ± = Standard Deviation.

Extraction method	Time (min)	Anneal temperature (°C)
Qiagen column	13.7 ± 0.8	79.8 ± 0.2
Chelex	15.0 ± 4.5	80.2 ± 0.3
HotShot	16.0 ± 2.0	80.3 ± 0.4
Xtract	14.6 ± 0.7	80.2 ± 0.2
QuickExtract	14.5 ± 2.7	80.1 ± 0.3
ddH_2_O	17.2 ± 2.1	80.3 ± 0.2

Overall, when compared by two-tailed, equal variance student t-test, there was a marginally significant difference between column extractions and HotShot (*p* = 0.048) and significant difference for column and water (*p* = 0.01), whilst water extractions were also significantly slower than Xtract, XT (*p* = 0.02) and QuickExtract, QE (*p* = 0.04). The widest variation in run times was recorded for Chelex (15.0 ± 4.5 min).

Overall, there was no difference between annealing temperatures for all methods, averaging at 80.1 ± 0.2 °C. All six of the DNA extraction methods we tested provided sufficient quality and quantity of DNA to perform the APL LAMP assay (Table 3; Fig. 3e, f). Three of the crude DNA extraction methods (QE, XT and ddH_2_O) were found suitable for near-field use, amplifying APL DNA within 12 to 18 min (Table 3).

## Discussion

Existing laboratory-based methods for APL molecular identification are available but are relatively slow as they involve obtaining DNA sequences for individual specimens. Due to the ambiguous morphology of the grasshopper nymphs, which often look very similar between numerous species found in the same habitat^4–6^, species identification is often achieved by engaging highly trained entomologists to perform morphological diagnostics^4–6^. However, this results in limited capacity to handle large numbers of specimens per day, thus potentially slowing biocontrol responses^4–6^. Additionally, identification of the incomplete specimens, eggs and immature stages is even more difficult, or impossible, by morphological means^4^.

In this study we developed a simple new LAMP assay to rapidly and accurately identify APL. Quick species identification of APL in nymphal stages is the cornerstone of locust management responses, which aim to minimise extensive damage to crops and pastures^4^. To aid and speed up locust diagnostics, we designed a primer set consisting of six primers (F3, B3, FIP and BIP and Loop primers), targeting a short section of the APL mitochondrial Cytb locus which enables species-specific amplification of the target gene region using LAMP to be completed in under half an hour. We coupled this assay with a simple rapid DNA extraction method enabling the complete assay, from submitted specimen to reliable ‘yes/no’ identification, to be performed within an hour.

Multiple DNA extraction methods were evaluated for their suitability for the APL LAMP assay. As expected, laboratory-grade Qiagen column extractions produced the cleanest DNA and the fastest LAMP amplification times, however this method requires specialised additional techniques and equipment such as centrifugation, relatively expensive reagents, and laboratory infrastructure, making it unsuitable for field deployment. In a surveillance triaging situation, the requirement for simple, quick extraction methods outweighs production of high-quality stabilised DNA extracts. The crude extraction methods tested — Xtract, QuickExtract (QE), HotShot, Chelex, and ddH₂O (water) — all generated sufficient DNA for the LAMP assay, with water extractions being the slowest, whereas Xtract and QE performed comparably to clean column extractions. Chelex showed the widest result variation, therefore, reflecting reduced reliability. For field use, QE, Xtract, and water were deemed most suitable, requiring only a heat source (e.g., portable LAMP instrument) without any need for centrifugation. QE and Xtract offer the advantage of stabilising DNA for later storage, while water is the simplest and cheapest approach, but it provides lower consistency and slower performance. Temperature requirements for these methods are minimal — primarily short incubations at 65 to 98 °C — compatible with incubation in the portable LAMP instrument. Overall, we concluded that for field diagnostics, QuickExtract or Xtract provide the best balance of speed, reliability, and minimal equipment needs, making them the most practical choices for rapid APL identification.

Additionally, by only using a small fragment of the specimen for DNA extractions, as we have done here, the rest of the insect body/sample can be preserved as a reference voucher specimen for additional morphological examination and molecular follow up, if required.

As there is a need for reliable positive controls for LAMP assays, we designed and optimised a synthetic dsDNA (gBlock) for use as a positive control, as has been used in other LAMP assays^13–15^. Generally, gBlocks are stable molecules, providing large amounts of control DNA which, in practice, enables the use of a gBlock of a known concentration to be used for tracking the performance of LAMP assay across different runs, to both act as a positive control and monitor the integrity and performance of the LAMP reagents^13^. Here the synthetic DNA fragment has been designed to produce a different annealing temperature between the APL DNA and gBlock (~ 3 °C difference), producing two discrete annealing peaks, to allow accurate discrimination of positive amplification from APL genomic DNA compared with amplification from the synthetic positive control. Additionally, in a situation where you are testing a potentially poorly preserved specimens, it may be advised to test quality of the extracted DNA by running a secondary 18 S LAMP test we previously devised for this purpose^13^ to avoid false negatives.

The new APL LAMP assay was found to amplify all APL samples tested here (Sup. Table), although several of the archived DNA samples (VAITC 09344 to VAITC 09688) amplified late due to DNA degradation during prolonged storage. We evaluated the specificity of this LAMP assay by testing it against a panel of grasshopper species closely associated with APL, which are often found within the same habitats as APL^5,16^. These included multiple closely related *Austroicetes* species and the more distantly related *Phaulicridium vitattum*. DNA barcoding of the Cytb locus confirmed that the assay performed as expected and only produced positive LAMP amplification from APL samples. All species tested appear to the untrained eye superficially morphologically similar to APL (Fig. 1), especially at the nymphal stages^5,16^(Fig. 1b). Non-target species tested did not amplify even when the LAMP run times were extended to 35 min, providing confidence that this assay will quickly and accurately identify only APL. Additionally, we have generated the first Cytb DNA barcode reference DNA sequences for 87 grasshopper species to assist with the future APL molecular diagnostics (Table 2).

Overall, our novel LAMP molecular identification assay provides a new tool to aid rapid and accurate identification of APL from all life stages and will likely prove most useful for identification of nymphs (and potentially eggs, not tested here). The speed of operation and accurate identification results generated will enable this test to be used at large scale to assist with control of APL, with positive or negative results available within an hour. The confidence in APL species identification that this method offers, and its simple use makes it highly suitable for early accurate diagnosis of this locust pest. This novel APL LAMP test is a new aid for APL field management and incursion response enabling rapid decision making and targeted management of locust populations before they swarm and spread causing widespread damage to the crops and grassland environments.

## Materials and methods

### Specimens examined

To enable testing the specificity of the new APL LAMP assay we generated a panel of reference DNA sequences of APL and ten other related Australian (Acrididae) grasshopper species, especially focusing on the closely related genus *Austroicetes*. This suite of native grasshoppers is commonly found in the same field environments as APL^1,5^ making morphological identification much more complex when immature stages are investigated. Samples of the target species (APL) and non-target grasshoppers were obtained from archived DNA (from 2006 to 2019) associated with two previous APL outbreaks and freshly collected field material (January 2025) from the Swifts Creek region in Victoria, as well as from *Austroicetes* specimens provided by Climatic and Metabolic Ecology laboratory (collected 2020/2021) (Sup. Table). A total of 87 grasshopper specimens were used for developing and testing this assay, including 39 specimens of APL, and multiple species of *Austroicetes*, the closest relative of APL (Figs. 1 and 4, Sup. Table). Identification of APL specimens and a non-target species were confirmed by DNA barcoding a section of the mitochondrial Cytb locus, following standard laboratory procedures (see below). The DNA barcode reference sequences generated in this study have been submitted to GenBank, accession numbers PX315616 – PX315702 (Table 2).

### DNA extraction

We tested 6 different DNA extraction methods comprising laboratory-based methods using Qiagen column extraction method, producing clean DNA extraction as well as quick DNA extraction methods for use in the field.

#### Laboratory-based DNA extraction methods

DNA was extracted from one of the middle locust legs, using a DNeasy Blood and Tissue extraction kit (Qiagen), following the manufacturers protocol. These samples provided laboratory grade “clean” DNA preparations for developing and optimising this LAMP assay, for testing the sensitivity of this assay, for DNA barcoding species identification. We used column extraction method destructively which yielded high quality “clean” DNA. All clean (laboratory prepared) DNA samples were quantified for DNA concentration using a Qubit 2.0 Fluorometer (Invitrogen, Life Technologies, Australia) following manufacturers protocol. Sampling only one leg enabled the preservation of the specimens as vouchers for morphological examination, and accessioning into insect reference collections.

#### Near-field compatible DNA extraction method

##### QuickExtract DNA extraction

For potential near-field use crude DNA extracts were prepared using the QuickExtract (QE) DNA extraction solution 1.0 (Epicentre, USA). Known APL samples were used to optimise the QE method. Fifty microlitres of QE were used for the DNA extracts prepared from a single 2 mm^2^ slice of the locust leg samples. The Genie strip was placed in the Genie III machine, used as an incubator for DNA extraction: 65 °C for 6 min, followed by 2 min at 98 °C, followed by > 1 min incubation on ice^12^.

##### Chelex DNA extraction

Chelex: Chelex 100 (BIO-RAD), is a chelating resin developed for DNA extraction use suitable for PCR. Fifty microlitres of a 5% (w/v) Chelex suspension in molecular grade water, with the addition of 5 µL proteinase K was prepared. We used single use disposable scalpel blade to slice approximately 2 mm^2^ locust leg and added it into the Chelex solution, then incubated it at 50 °C for 30 min followed by 98 °C for 2 min (Total run time = 32 min). Samples were centrifuged for 2 min at 17 000 rpm to ensure resin beads were absent in the suspension, prior to using as template DNA for the APL LAMP assay.

##### Xtract DNA extraction

Xtract: Additional DNA extracts were prepared from single 2 mm^2^ slice of the locust leg using Xtract (Xt) DNA extraction solution (GeneWorks, AU), using 50 µL of Xtract buffer, following published protocols^12^.

##### HotShot DNA extraction

HotShot: the DNA extracts were prepared from a single 2 mm^2^ slice of the locust leg using a HotShot “HS6” protocol^17^ modified and published by Agarwal et al. (2020).

##### ddH_2_O DNA extraction

Water can also be used as a DNA extraction medium for LAMP assays^18^. We tested extraction of DNA using just plain double distilled laboratory H_2_0. Extractions were prepared from single 2 mm^2^ slice of the locust leg and placed into 50 µL of dd H_2_0 which was extracted in OptiGene tube stirps (OptiGene, UK), using Genie III LAMP machine (GeneWorks, AU), keeping the strip at 95 °C for 5 min.

All these DNA extraction methods were tested using preserved specimen of APL (VAITC 9713). The near-field extraction protocols were performed in OptiGene tube stirps (OptiGene, UK), using Genie III LAMP machine (GeneWorks, AU). One µL of DNA extraction was used as a template in each of the LAMP reaction tests (Table 3).

### APL dataset, primer design and LAMP assay optimisation

The development of this assay did not use the standard Cytochrome Oxidase I (COI) DNA barcoding region, as initial work revealed nuclear pseudogene/s (numt/s) were present for this locus in APL, and therefore not being able to generate accurate COI DNA sequences for this species (Blacket *unpublished*). An alternative mitochondrial locus, Cytb, which has previously been used for identification of grasshopper species^7^ was targeted for the new LAMP assay. Cytb DNA sequences of APL and multiple *Austroicetes* species were obtained and aligned to use for designing new LAMP primers specific for APL (Fig. 2).

Six novel LAMP primers were manually designed by eye to target a region of Cytb (Table 1). Complete sets of LAMP primers were analysed together to calculate predicted GC content (%), predicted primer melting temperatures (Tm), and detect potential primer dimer interactions, using the ThermoFisher Scientific Multiple Primer Analyzer tool (www.thermofisher.com). Primers were synthesised by Sigma (Australia).

LAMP primers (Table 1) were developed to target the APL Cytb locus (Fig. 2). Six primers are used in the APL LAMP assay, two inner primers (FIP and BIP) and two outer primers (F3 and B3). The addition of loop primers (Floop and Bloop) makes LAMP reaction more rapid to enable amplification of positive DNA in 50% less time^19^. We tested 1:4:2 and 1:6:3 and 1:8:4 primer ratios (F3/B3: FIP/BIP: Floop/Bloop), following a primer-optimisation published protocol^11^. The final optimised primer mix ratio 1:4:2 was prepared by adding the specified amount of each of the six primers: for a 100 µL volume of primer mix 1:4:2 (F3/B3: FIP/BIP: Floop/Bloop) we added: 10 µL each of F3/B3 (10 µM), 4 µL each of FIP/BIP (100 µM), 2 µL each of Floop/Bloop (100 µM) and 68 µL of Ultrapure water (Invitrogen, Australia), bringing final primer concentration to 0.4 µM, 1.6 µM and 0.8 µM, respectively.

Each LAMP reaction mix was made by adding 10 µL of primer mix to 14 µL of Isothermal Master Mix (ISO-004, OptiGene, UK) and 1 µL of template DNA into each well of the Genie strip (25 µL total reaction volume). Each run included a positive control (gBlock, Fig. 4), a no-template negative control, and six test samples. All LAMP assays were run in the Genie III at 65 °C for 35 min followed by an annealing curve analysis from 98 °C to 73 °C with ramping at 0.05 °C/s. The total run time being approximately 35 min.

The amplification and anneal derivative curves were visualised on the Genie lll screen to ensure that amplification occurred as expected. Positive results were further confirmed through performing the annealing step which resulted in a single product peak at a specific temperature (Fig. 3b, d, f). The negative control (NTC) remains a flat line. Results were analysed using a PC version of the software Genie Explorer version 2.0.7.11 in the blue channel.

### Analytic sensitivity of the APL LAMP assay

A four-fold serial dilution (1:4) of clean DNA extract of APL (specimen VAITC 9303) (clean, Qiagen column DNA extraction) DNA was prepared using Ultrapure water (Invitrogen, Life Technologies, Australia). The starting DNA concentration was quantified using a Qubit 2.0 Fluorometer (Invitrogen, Life Technologies, Australia) following the manufacturers protocol. The DNA sample was serially diluted up to 8 dilutions (Fig. 3c, d), from 5.3 ng/µl to 3.5 × 10 − 4 ng/µL (1:1 to 1:256) and used as template for the APL LAMP assay.

### Analytic specificity of the APL LAMP assay

**DNA barcoding identification and sequence analysis**.

Identifications of all grasshopper specimens included in this study were performed through DNA barcoding of a 388 bp fragment of the Cytb locus. Polymerase chain reaction (PCR) was performed using the Bioline MyFi DNA Polymerase kit (Meridian Bioscience, Ohio, United States of America) using 2.5 µL of DNA template, 5 µL of polymerase buffer, 0.5 µL of MyFi polymerase and 1 µL each for the two primers (10 mM) in a 25 µL final reaction volume. We used the Cytb gene primer pair Ghop_mtd26_Cytb-F 5’- TATGTACTACCATGAGGACAAATATC-3’ ^7^, and Ghop_Cytb-R 5’-GCAAATARGAAATATCATTCWGGTTG-3’ (designed for this study based on the locust mtGenome, GenBank X80245). The PCR was run with the following cycling conditions: an initial 5-minute denaturation at 95 °C, followed by 35 cycles of denaturation at 95 °C for 45 s, annealing at 50 °C for 30 s and extension at 72 °C for 30 s, followed by a final extension at 72 °C for 7 minutes. PCR amplification was verified on a 1% w/v agarose gel.

All amplified PCR products were purified and sequenced both ways commercially by AGRF (Melbourne, Australia), as Sanger DNA sequences. The quality of DNA sequences obtained was confirmed by eye, and consensus DNA sequences were aligned for comparison using Mega11 software^20^. A Neighbour Joining tree of relationships between species was generated in Mega11, with 100 bootstraps.

### Evaluation of a gBlock gene fragment for APL LAMP assay

A 252 bp gBlock dsDNA fragment (Integrated DNA Technologies, Iowa, USA) was designed for use as synthetic DNA positive control for the APL LAMP assay. This synthetic fragment consisted solely of concatenated LAMP primer sites separated by runs of “cctcc”, to increase the overall Tm of the fragment (Table 1). To evaluate detection sensitivity, the copy number and a ten-fold serial dilution (1:10) of the gBlock was prepared as previously published^14^. Sensitivity of the LAMP assay was tested using the serially diluted (1 × 10^8^ to 1 × 10 copies/µL) of gBlock in the Genie III, following the APL LAMP assay conditions mentioned above. Following this test, another LAMP run was conducted to determine the best dilution for gBlock to be used as a positive control in LAMP assays. The same four-fold serial dilution of APL DNA (VAITC 9303) (5.27ng/µL to 0.000353 ng/µL) was used as template to compare amplification time with one million copies (1 × 106 copies/µL) of gBlock (Fig. 3a, b).

## Supplementary Information

Below is the link to the electronic supplementary material.

Supplementary Material 1

## Data Availability

All DNA sequences generated for this study were uploaded on GenBank, with accession numbers PX315616 – PX315702.

## References

[1] APLC. Department of Agriculture, Fisheries and Forestry; Australian Plague Locust Commission. https://www.agriculture.gov.au/biosecurity-trade/pests-diseases-weeds/locusts (2024).

[2] DAFF. Australian plague locust; Department of Agriculture, Fisheries and Forestry. https://www.agriculture.gov.au/biosecurity-trade/pests-diseases-weeds/locusts/about/australia (2022).

[3] Law, S. R. et al. Molecular profiling of the Australian plague locust pathobiome reveals a microbial driver of population suppression. *J. Invertebr. Pathol.* **213**, 108402 (2025). 40712665 10.1016/j.jip.2025.108402

[4] Agriculture Victoria. Australian plague locusts, Agriculture Victoria. https://agriculture.vic.gov.au/biosecurity/pest-insects-and-mites/priority-pest-insects-and-mites/plague-locusts (2025).

[5] Rentz, D. C. F., Lewis, R. C., Su, Y. N. & Upton, M. S. *A Guide to Australian Grasshoppers and Locusts* (Natural History Publications (Borneo) Sdn. Bhd, 2003).

[6] Agriculture Victoria. Australian Plague Locust Field Surveillance Guide. https://agriculture.vic.gov.au/biosecurity/pest-insects-and-mites/priority-pest-insects-and-mites/plague-locusts/australian-plague-locust-biology-and-behaviour (2025).

[7] Fries, M., Chapco, W. & Contreras, D. A molecular phylogenetic analysis of the Oedipodinae and their intercontinental relationships. *J. Orthoptera Res.* **16**, 115–125 (2007).

[8] Song, H., Moulton, M. J. & Whiting, M. F. Rampant nuclear insertion of mtDNA across diverse lineages within Orthoptera (Insecta). *PLoS ONE* **9**(10), e110508 (2014). 10.1371/journal.pone.0110508 PMC4204883 25333882

[9] Hebert, P., Cywinska, A., Ball, S. L. & deWaard, J. Biological identification through DNA barcodes. *Proc. R. Soc. Lond. B. Biol. Sci.* **270**, 313–321 (2003). 10.1098/rspb.2002.2218 PMC1691236 12614582

[10] Notomi, T. Loop-mediated isothermal amplification of DNA. *Nucleic Acids Res.* **28**, e63 (2000). 10871386 10.1093/nar/28.12.e63 PMC102748

[11] Blacket, M. J. et al. A LAMP assay for detection of *Bactrocera tryoni* Queensland fruit fly (Diptera: Tephiritidae). *Sci. Rep.* **10**, 9554 (2020). 32533005 10.1038/s41598-020-65715-5 PMC7293347

[12] Agarwal, A., Cunningham, J. P., Valenzuela, I. & Blacket, M. J. A diagnostic LAMP assay for the destructive grapevine insect pest, phylloxera (*Daktulosphaira vitifoliae*). *Sci. Rep.* **10**, 1–10 (2020). 33277555 10.1038/s41598-020-77928-9 PMC7718921

[13] Rako, L. et al. A LAMP (Loop-mediated isothermal amplification) test for rapid identification of Khapra beetle (*Trogoderma granarium*). *Pest Manag. Sci.* **77**(12), 5509–5521 (2021). 34363302 10.1002/ps.6591 PMC9290502

[14] Agarwal, A. et al. A diagnostic LAMP assay for rapid identification of an invasive plant pest, fall armyworm *Spodoptera frugiperda* (Lepidoptera: Noctuidae). *Sci. Rep.* **12**(1), 1116 (2022). 35064176 10.1038/s41598-021-04496-x PMC8782856

[15] Rako, L. et al. LAMP (Loop-mediated isothermal amplification) assay for rapid identification of Varroa mites. *Sci. Rep.* **13**, 11931 (2023). 37488147 10.1038/s41598-023-38860-w PMC10366197

[16] Key, K. H. L. *The Taxonomy, Phases, and Distribution of the Genera Chortoicetes Brunn. and Austroicetes Uv. (Orthoptera: Acrididae)* (Division of Entomology, Commonwealth Scientific and Industrial Research Organization, 1954).

[17] Zieritz, A. et al. Development and evaluation of HotShot protocols for cost- and time-effective extraction of PCR-ready DNA from single freshwater mussel larvae (Bivalvia: Unionida). *J. Molluscan Stud.* **84**, 198–201 (2018).

[18] Sabahi, S., Fekrat, L., Zakiaghl, M. & Moravej, G. H. Loop-mediated isothermal amplification combined with PCR for rapid identification of the Ethiopian fruit fly (Diptera: Tephritidae). *Neotrop. Entomol.* **47**, 96–105 (2018). 28478540 10.1007/s13744-017-0522-2

[19] Soroka, M., Wasowicz, B. & Rymaszewska, A. Loop-mediated isothermal amplification (LAMP): The better sibling of PCR?. *Cells* **29**(10(8)), 1931 (2021). 10.3390/cells10081931 PMC8393631 34440699

[20] Tamura, K., Stecher, G. & Kumar, S. MEGA11: Molecular evolutionary genetics analysis version 11. *Mol. Biol. Evol.* **38**, 3022–3027 (2021). 33892491 10.1093/molbev/msab120 PMC8233496

