# Supplementary material for: Loop-mediated isothermal amplification (LAMP) assay for identification of Australian Plague Locust (APL), Chortoicetes terminifera (Walker, 1870)
Source: Sci Rep. 2026 May 21;16:19352. doi: 10.1038/s41598-026-50241-7 (PMC13287623; doi:10.1038/s41598-026-50241-7)
Supplement: Supplementary file 1 — Supplementary Material 1 [file 41598_2026_50241_MOESM1_ESM.docx]

**Supplementary Table:** List of the 8 grasshopper specimens tested using the APL (*Chortoicetes terminifera*) LAMP assay. Grey shading indicates APL species (target taxa). DNA extraction: Qiagen column (column), HotSHot (HT), Xtract (XT), QuickExtract (QE), Chelex, ddH_2_O (H_2_O). The symbol “x” has been used when no amplification was recorded.

| **Specimen #** | **life stage** | **Speies** | **DNA extraction method** | **LAMP Time (min)** | **LAMP °C** | **GenBank Accession** |
| --- | --- | --- | --- | --- | --- | --- |
| VAITC09668 | Archived DNA | *Acrida conica* | Column | x | x | PX315616 |
| WAR7153 | Adult | *Austroicetes* sp. 1 | Column | x | x | PX315617 |
| WAR7155 | Adult | *Austroicetes* sp. 1 | Column | x | x | PX315618 |
| WAR7158 | Adult | *Austroicetes* sp. 1 | Column | x | x | PX315619 |
| WAR7159 | Nymph | *Austroicetes* sp. 1 | Column | x | x | PX315620 |
| WAR8264 | Adult | *Austroicetes* sp. 1 | Column | x | x | PX315621 |
| WAR8285 | Adult | *Austroicetes* sp. 1 | Column | x | x | PX315622 |
| WAR8300b | Nymph | *Austroicetes* sp. 1 | Column | x | x | PX315623 |
| WAR8303 | Adult | *Austroicetes* sp. 1 | Column | x | x | PX315624 |
| WAR8305 | Adult | *Austroicetes* sp. 1 | Column | x | x | PX315625 |
| WAR8327 | Adult | *Austroicetes* sp. 1 | Column | x | x | PX315626 |
| WAR8332 | Adult | *Austroicetes* sp. 1 | Column | x | x | PX315627 |
| WAR8349 | Adult | *Austroicetes* sp. 1 | Column | x | x | PX315628 |
| VAITC09213 | Archived DNA | *Austroicetes* sp. 2 | Column, HS | x | x | PX315629 |
| VAITC09617 | Archived DNA | *Austroicetes* sp. 2 | Chelex | x | x | PX315630 |
| VAITC09699 | Archived DNA | *Austroicetes* sp. 2 | Column | x | x | PX315631 |
| VAITC09735 | Archived DNA | *Austroicetes* sp. 2 | Column | x | x | PX315632 |
| WAR8257 | Adult | *Austroicetes* sp. 2 | Column | x | x | PX315633 |
| WAR8283 | Adult | *Austroicetes* sp. 2 | Column | x | x | PX315634 |
| WAR8306 | Adult | *Austroicetes* sp. 2 | Column | x | x | PX315635 |
| WAR8320 | Adult | *Austroicetes* sp. 2 | Column | x | x | PX315636 |
| WAR7156 | Adult | *Austroicetes* sp. 3 | Column | x | x | PX315637 |
| WAR7157 | Adult | *Austroicetes* sp. 3 | Column | x | x | PX315638 |
| VAITC09307 | Archived DNA | *Austroicetes* sp. 4 | Column | *x* | x | PX315639 |
| VAITC09308 | Archived DNA | *Austroicetes* sp. 4 | Column | *x* | x | PX315640 |
| VAITC10665 | Archived DNA | *Austroicetes* sp. 4 | Chelex | x | x | PX315641 |
| VAITC10666 | Archived DNA | *Austroicetes* sp. 4 | Column, Chelex | x | x | PX315642 |
| WAR7152 | Adult | *Austroicetes* sp. 4 | Column | x | x | PX315643 |
| WAR7163 | Nymph | *Austroicetes* sp. 4 | Column | x | x | PX315644 |
| VAIC085900 | Adult | *Chortoicetes terminifera* | Column, H2O | 18.35 | 79.64 | PX315645 |
| VAIC086902 | Nymph | *Chortoicetes terminifera* | Column, H2O | 15.15 | 79.67 | PX315646 |
| VAIC086904 | Nymph | *Chortoicetes terminifera* | Column, H2O | 18.15 | 79.54 | PX315647 |
| VAIC085936 | Adult | *Chortoicetes terminifera* | Column, H2O | 22.75 | 79.99 | PX315648 |
| VAIC085945 | Adult | *Chortoicetes terminifera* | Column, H2O | 23 | 79.68 | PX315649 |
| VAIC085946 | Adult | *Chortoicetes terminifera* | Column, H2O | 29.52 | 79.58 | PX315650 |
| VAITC 09713 | Nymph | *Chortoicetes terminifera* | Column, Chelex, HS, XE, QE, H_2_O | 15.18 | 80.12 | PX315651 |
| VAITC09303 | Archived DNA | *Chortoicetes terminifera* | Column | *15.75* | 79.97 | PX315652 |
| VAITC09304 | Archived DNA | *Chortoicetes terminifera* | Column | *16.07* | 80.14 | PX315653 |
| VAITC09335 | Archived DNA | *Chortoicetes terminifera* | Column | *15.97* | 79.93 | PX315654 |
| VAITC09339 | Archived DNA | *Chortoicetes terminifera* | Column | *18.1* | 79.93 | PX315655 |
| VAITC09340 | Archived DNA | *Chortoicetes terminifera* | Column | *18.45* | 79.99 | PX315656 |
| VAITC09343 | Archived DNA | *Chortoicetes terminifera* | Column | *17.3* | 79.51 | PX315657 |
| VAITC09344 | Archived DNA | *Chortoicetes terminifera* | Column | *late amplification* | 79.28 | PX315658 |
| VAITC09345 | Archived DNA | *Chortoicetes terminifera* | Column | *15.93* | 79.65 | PX315659 |
| VAITC09607 | Archived DNA | *Chortoicetes terminifera* | Chelex | *late amplification* | 79.33 | PX315660 |
| VAITC09609 | Archived DNA | *Chortoicetes terminifera* | Chelex | *late amplification* | 79.63 | PX315661 |
| VAITC09610 | Archived DNA | *Chortoicetes terminifera* | Chelex | *late amplification* | 79.73 | PX315662 |
| VAITC09612 | Archived DNA | *Chortoicetes terminifera* | Chelex | *late amplification* | 79.65 | PX315663 |
| VAITC09613 | Archived DNA | *Chortoicetes terminifera* | Chelex | *late amplification* | 79.69 | PX315664 |
| VAITC09614 | Archived DNA | *Chortoicetes terminifera* | Chelex | late amplification | 79.63 | PX315665 |
| VAITC09688 | Archived DNA | *Chortoicetes terminifera* | Column | late amplification | 79.66 | PX315666 |
| VAITC09691 | Archived DNA | *Chortoicetes terminifera* | Column | 19.73 | 79.8 | PX315667 |
| VAITC09692 | Archived DNA | *Chortoicetes terminifera* | Column | 18.73 | 79.79 | PX315668 |
| VAITC09693 | Archived DNA | *Chortoicetes terminifera* | Column | 21.8 | 79.74 | PX315669 |
| VAITC09696 | Archived DNA | *Chortoicetes terminifera* | Column | 14 | 79.72 | PX315670 |
| VAITC09808 | Archived DNA | *Chortoicetes terminifera* | Column | 15.38 | 80 | PX315671 |
| VAITC09809 | Archived DNA | *Chortoicetes terminifera* | Column | 19.67 | 80 | PX315672 |
| VAITC09810 | Archived DNA | *Chortoicetes terminifera* | Column | 14.32 | 79.86 | PX315673 |
| VAITC09811 | Archived DNA | *Chortoicetes terminifera* | Column | 17.2 | 79.98 | PX315674 |
| VAITC09812 | Archived DNA | *Chortoicetes terminifera* | Column | 19.67 | 79.81 | PX315675 |
| VAITC09813 | Archived DNA | *Chortoicetes terminifera* | Column | 16.32 | 79.72 | PX315676 |
| VAITC09814 | Archived DNA | *Chortoicetes terminifera* | Column | 18.6 | 80.08 | PX315677 |
| WAR8300a | Nymph | *Chortoicetes terminifera* | Column | 16.28 | 80.3 | PX315678 |
| VAITC10660 | Archived DNA | Acrididae sp. 1 | Chelex | x | x | PX315679 |
| VAITC10661 | Archived DNA | Acrididae sp. 1 | Chelex | x | x | PX315680 |
| VAITC09341 | Archived DNA | *Oedipodinae* sp. 2 | Column | *x* | x | PX315681 |
| VAITC09817 | Archived DNA | *Oedipodinae* sp. 2 | Column | x | x | PX315682 |
| VAITC10656 | Archived DNA | *Oedipodinae* sp. 2 | Chelex | x | x | PX315683 |
| VAITC09337 | Archived DNA | *Oedipodinae* sp. 1 | Column | *x* | x | PX315684 |
| VAITC09305 | Archived DNA | *Phaulacridium vittatum* | Column | *x* | x | PX315685 |
| VAITC09618 | Archived DNA | *Phaulacridium vittatum* | Chelex | x | x | PX315686 |
| VAITC10653 | Archived DNA | *Phaulacridium vittatum* | Chelex | x | x | PX315687 |
| VAITC10654 | Archived DNA | *Phaulacridium vittatum* | Chelex | x | x | PX315688 |
| VAITC10657 | Archived DNA | *Phaulacridium vittatum* | Chelex | x | x | PX315689 |
| VAITC10658 | Archived DNA | *Phaulacridium vittatum* | Chelex | x | x | PX315690 |
| VAITC10659 | Archived DNA | *Phaulacridium vittatum* | Chelex | x | x | PX315691 |
| VAITC10662 | Archived DNA | *Phaulacridium vittatum* | Chelex | x | x | PX315692 |
| VAITC10663 | Archived DNA | *Phaulacridium vittatum* | Chelex | x | x | PX315693 |
| VAITC10664 | Archived DNA | *Phaulacridium vittatum* | Chelex | x | x | PX315694 |
| VAITC10668 | Archived DNA | *Phaulacridium vittatum* | Chelex | x | x | PX315695 |
| VAITC10669 | Archived DNA | *Phaulacridium vittatum* | Chelex | x | x | PX315696 |
| VAITC10673 | Archived DNA | *Phaulacridium vittatum* | Chelex | x | x | PX315697 |
| VAITC09697 | Archived DNA | *Praxibulus* sp. 1 | Column | x | x | PX315698 |
| VAITC09698 | Archived DNA | *Praxibulus* sp. 1 | Column | x | x | PX315699 |
| VAITC09734 | Archived DNA | *Praxibulus* sp. 1 | Column | x | x | PX315700 |
| VAITC10670 | Archived DNA | *Praxibulus* sp. 1 | Chelex | x | x | PX315701 |
| VAITC10671 | Archived DNA | *Praxibulus* sp. 1 | Chelex | x | x | PX315702 |
